# Supplementary material for: Genome-Wide Identification of GRAS Transcription Factors and Their Functional Analysis in Salt Stress Response in Sugar Beet
Source: Int J Mol Sci. 2024 Jun 28;25(13):7132. doi: 10.3390/ijms25137132 (PMC11241673; doi:10.3390/ijms25137132)
Supplement: Supplementary file 1 [file ijms-25-07132-s001.zip › Supplementary Figure-Xiaolin Hao.pptx]

## Slide 1
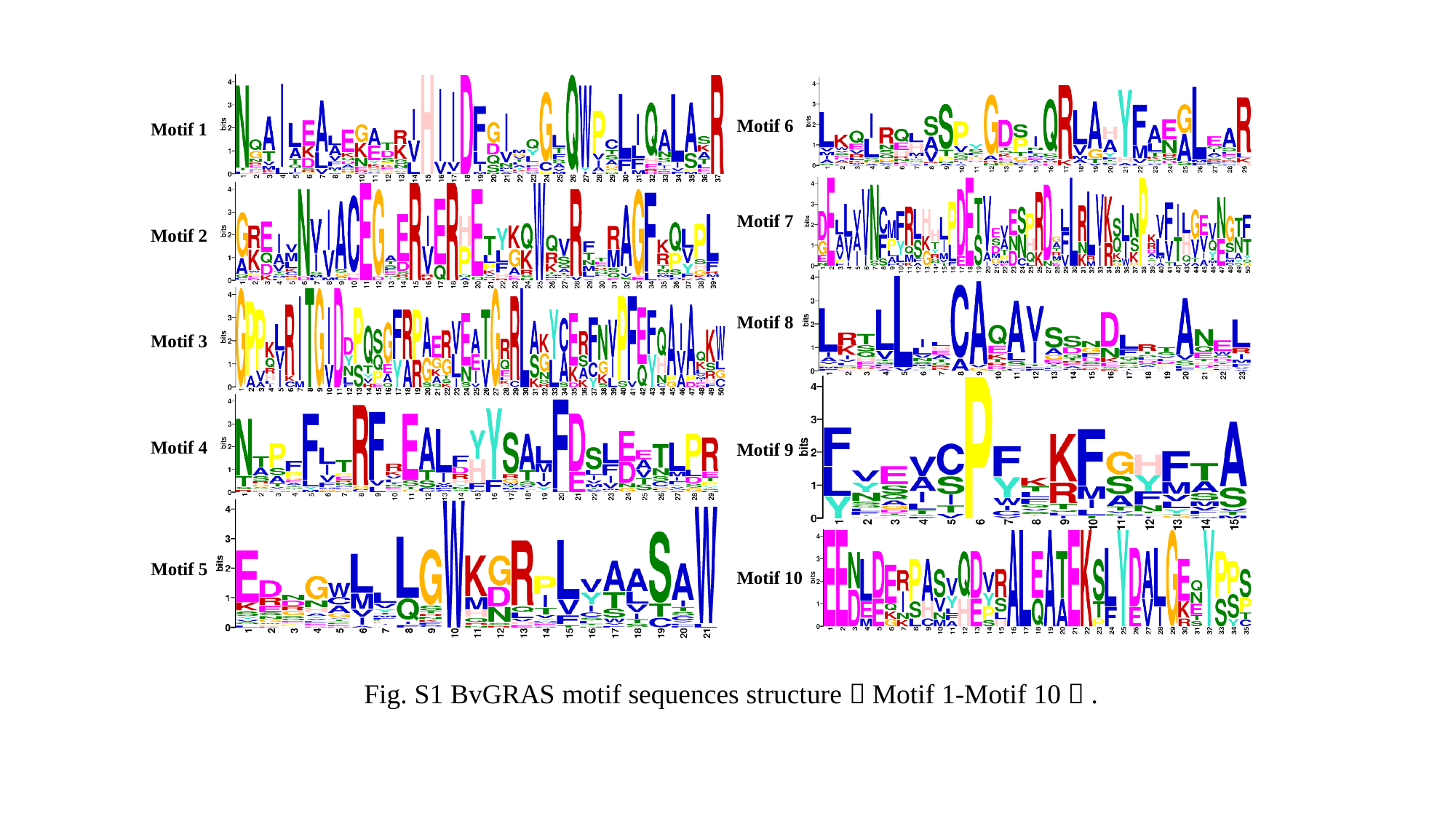

Motif 6
Motif 1
Motif 7
Motif 2
Motif 8
Motif 3
Motif 4
Motif 9
Motif 5
Motif 10
Fig. S1 BvGRAS motif sequences structure（Motif 1-Motif 10）.

## Slide 2
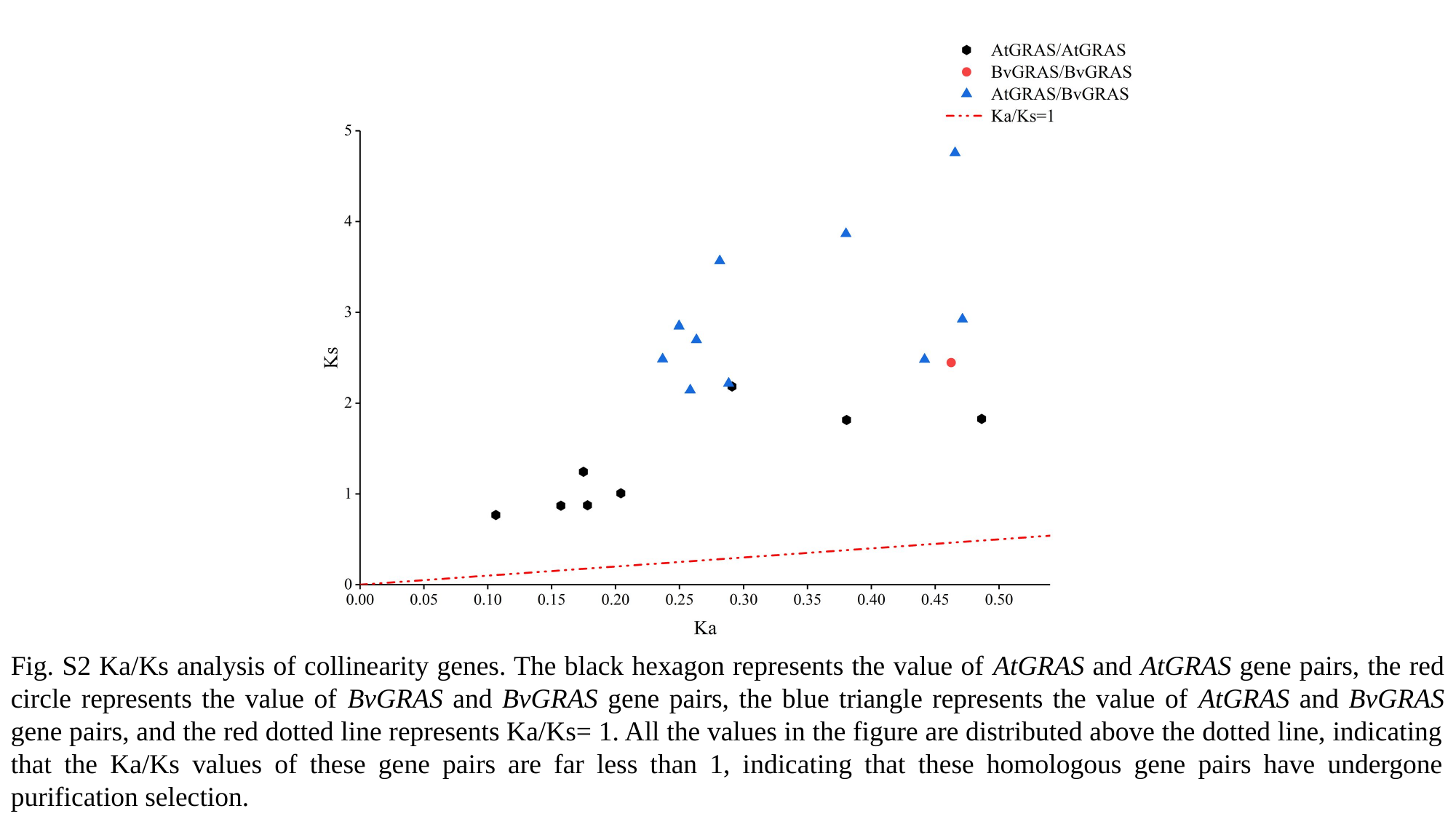

Fig. S2 Ka/Ks analysis of collinearity genes. The black hexagon represents the value of AtGRAS and AtGRAS gene pairs, the red circle represents the value of BvGRAS and BvGRAS gene pairs, the blue triangle represents the value of AtGRAS and BvGRAS gene pairs, and the red dotted line represents Ka/Ks= 1. All the values in the figure are distributed above the dotted line, indicating that the Ka/Ks values of these gene pairs are far less than 1, indicating that these homologous gene pairs have undergone purification selection.

## Slide 3
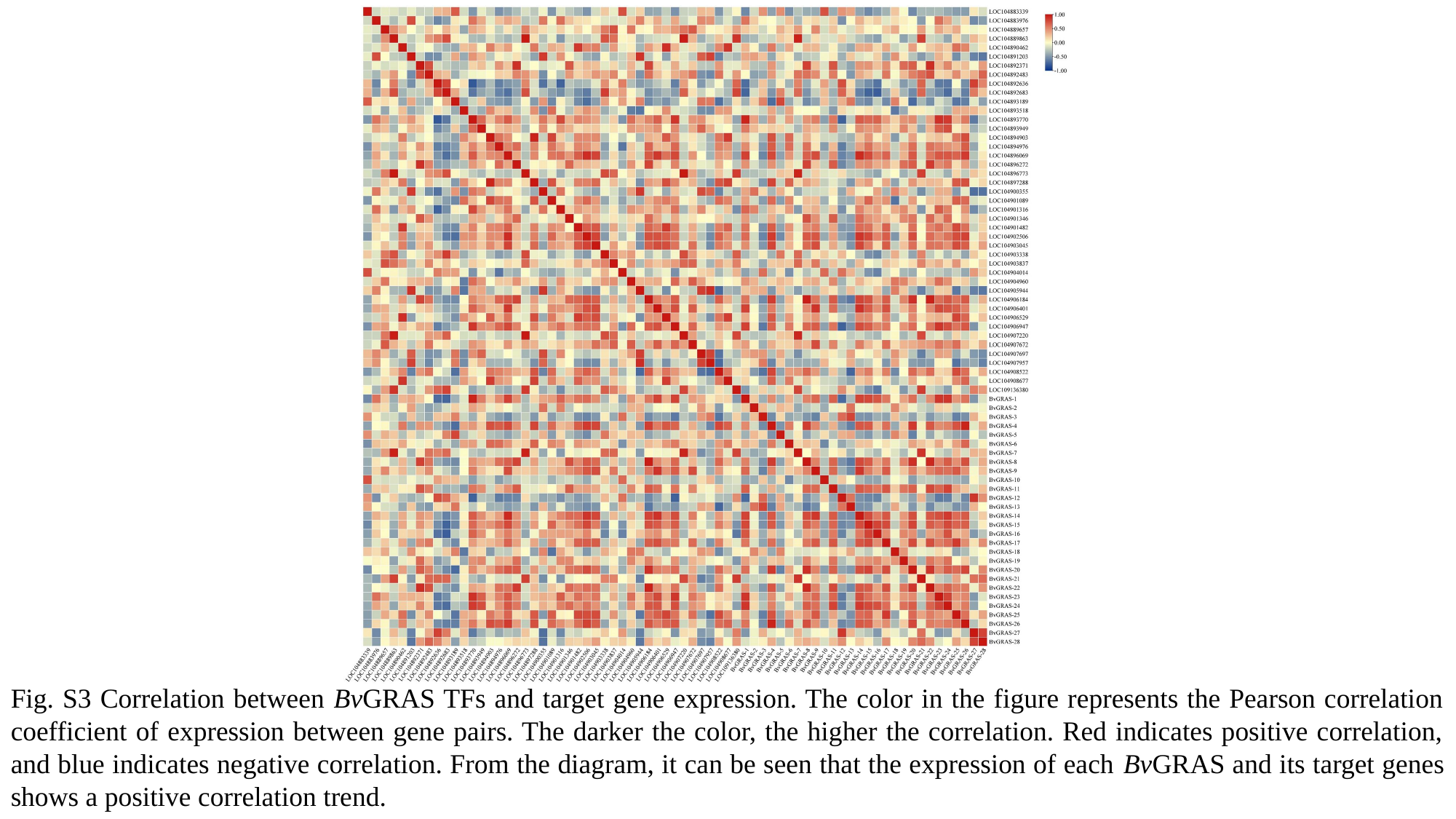

Fig. S3 Correlation between BvGRAS TFs and target gene expression. The color in the figure represents the Pearson correlation coefficient of expression between gene pairs. The darker the color, the higher the correlation. Red indicates positive correlation, and blue indicates negative correlation. From the diagram, it can be seen that the expression of each BvGRAS and its target genes shows a positive correlation trend.

## Slide 4
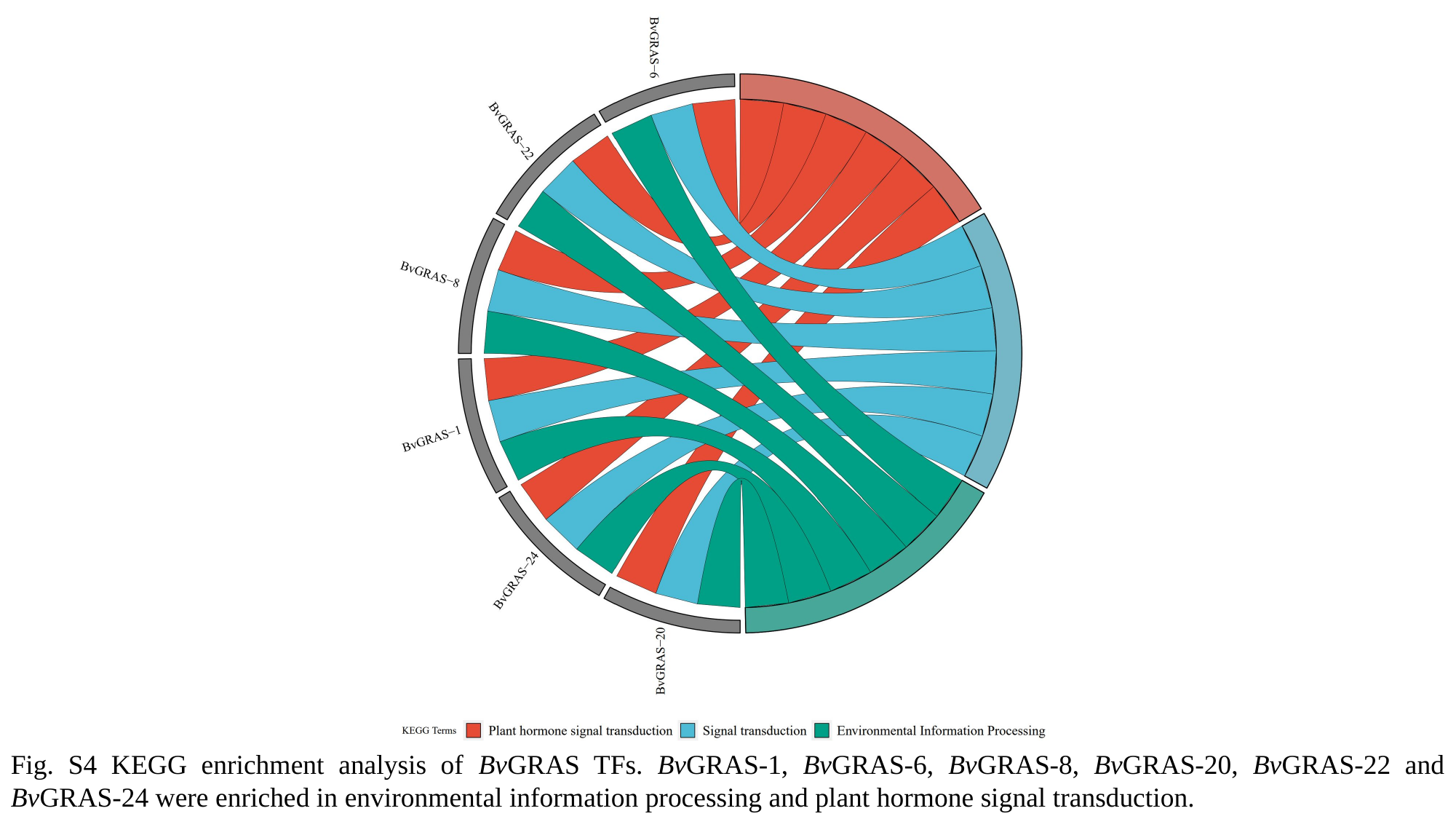

Fig. S4 KEGG enrichment analysis of BvGRAS TFs. BvGRAS-1, BvGRAS-6, BvGRAS-8, BvGRAS-20, BvGRAS-22 and BvGRAS-24 were enriched in environmental information processing and plant hormone signal transduction.

## Slide 5
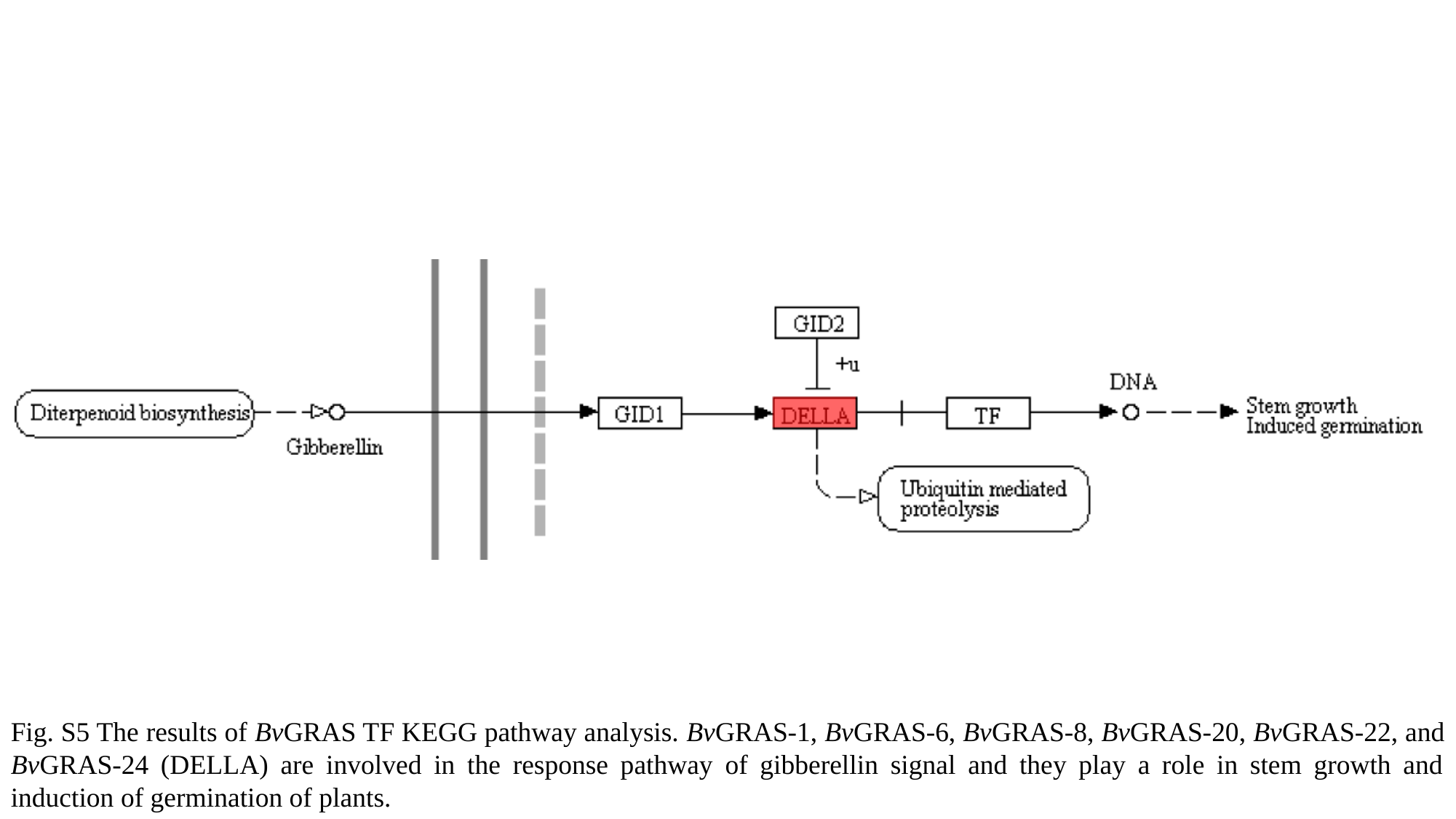

Fig. S5 The results of BvGRAS TF KEGG pathway analysis. BvGRAS-1, BvGRAS-6, BvGRAS-8, BvGRAS-20, BvGRAS-22, and BvGRAS-24 (DELLA) are involved in the response pathway of gibberellin signal and they play a role in stem growth and induction of germination of plants.

## Slide 6
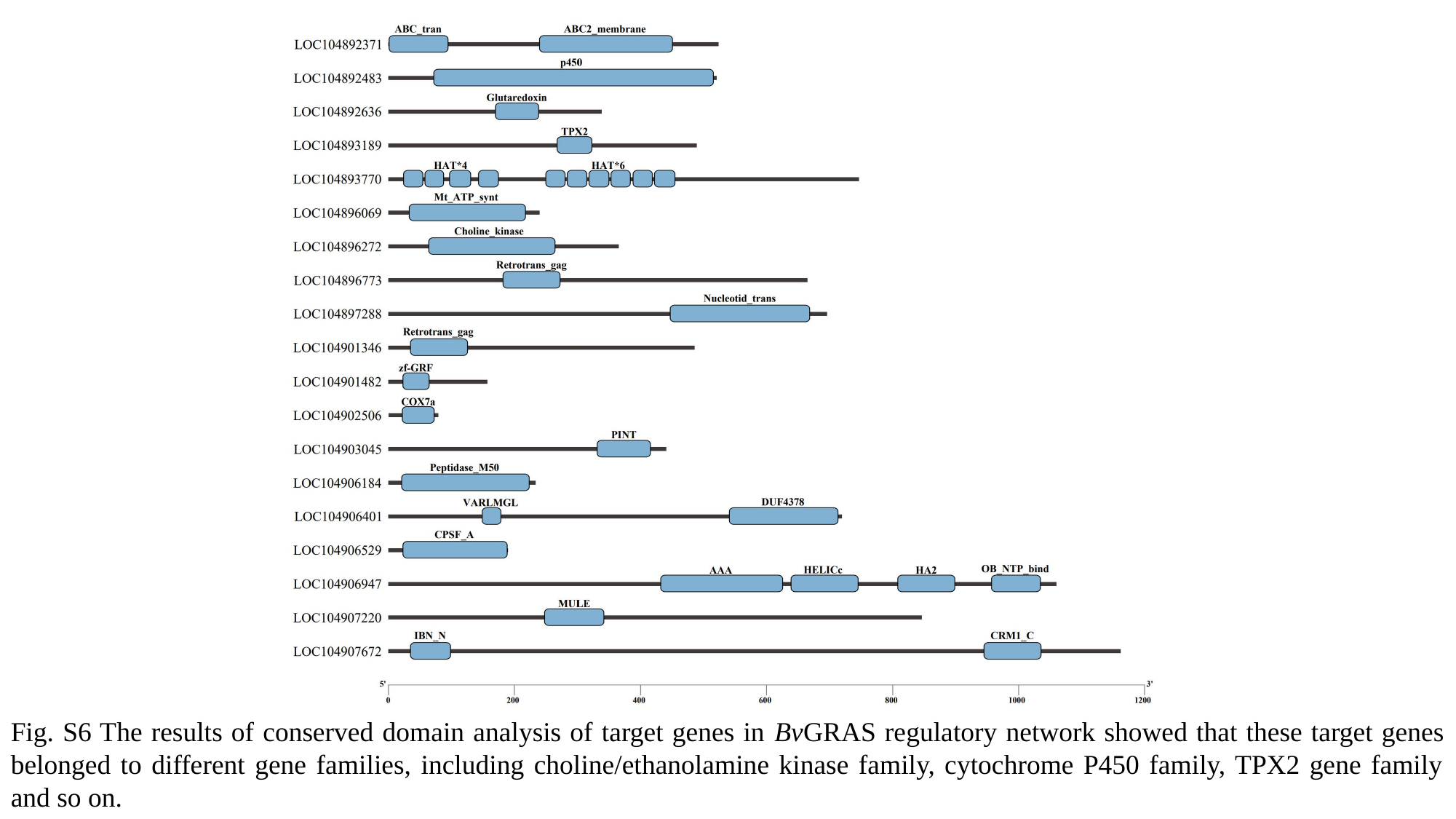

Fig. S6 The results of conserved domain analysis of target genes in BvGRAS regulatory network showed that these target genes belonged to different gene families, including choline/ethanolamine kinase family, cytochrome P450 family, TPX2 gene family and so on.
