# Supplementary figures and images for: Genome-Wide Identification of GRAS Transcription Factors and Their Functional Analysis in Salt Stress Response in Sugar Beet
Source: Int J Mol Sci. 2024 Jun 28;25(13):7132. doi: 10.3390/ijms25137132 (PMC11241673; doi:10.3390/ijms25137132)

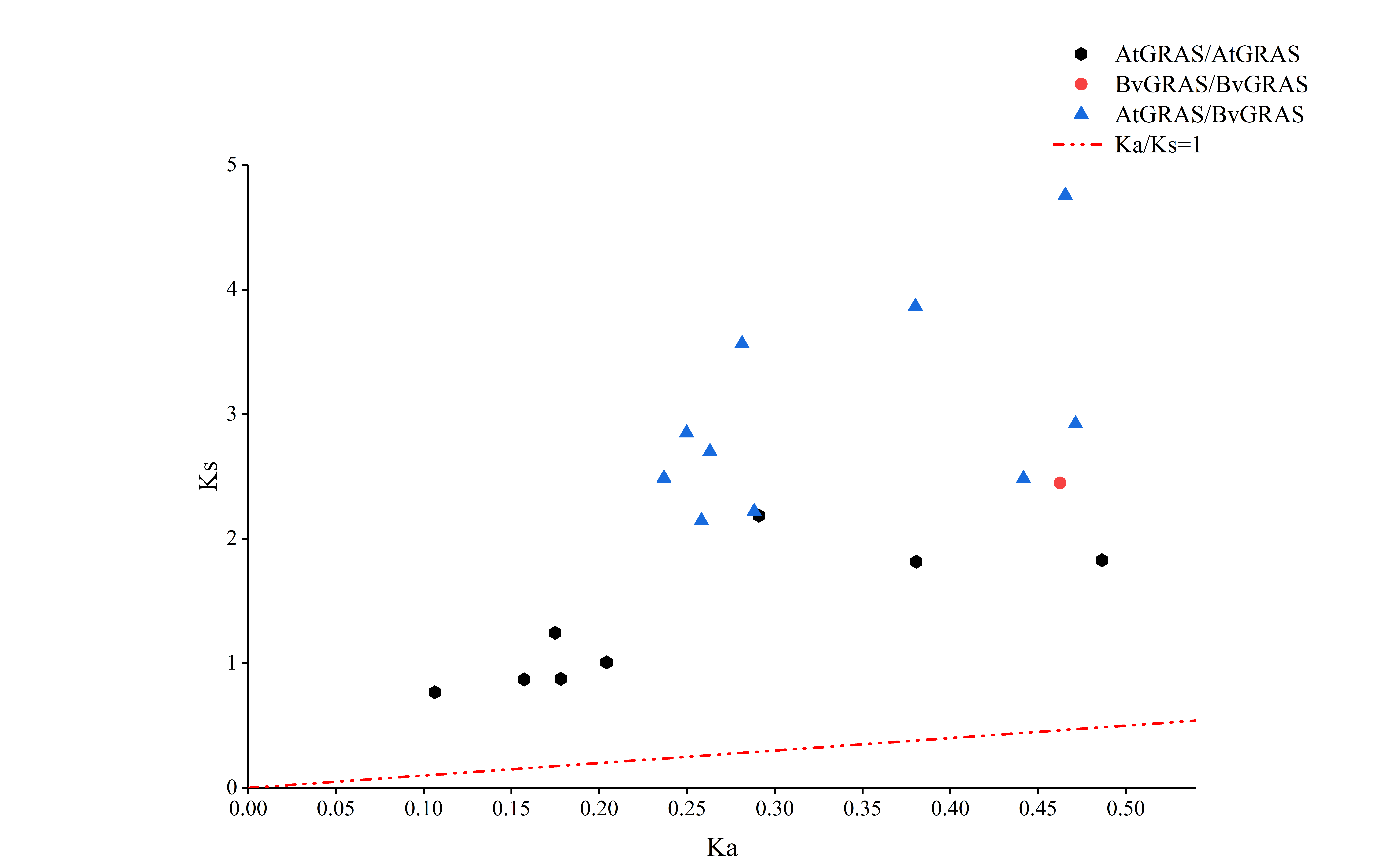

Supplement: Supplementary file 1 [file ijms-25-07132-s001.zip › Figure S2 KaKs analysis of collinearity genes.png]

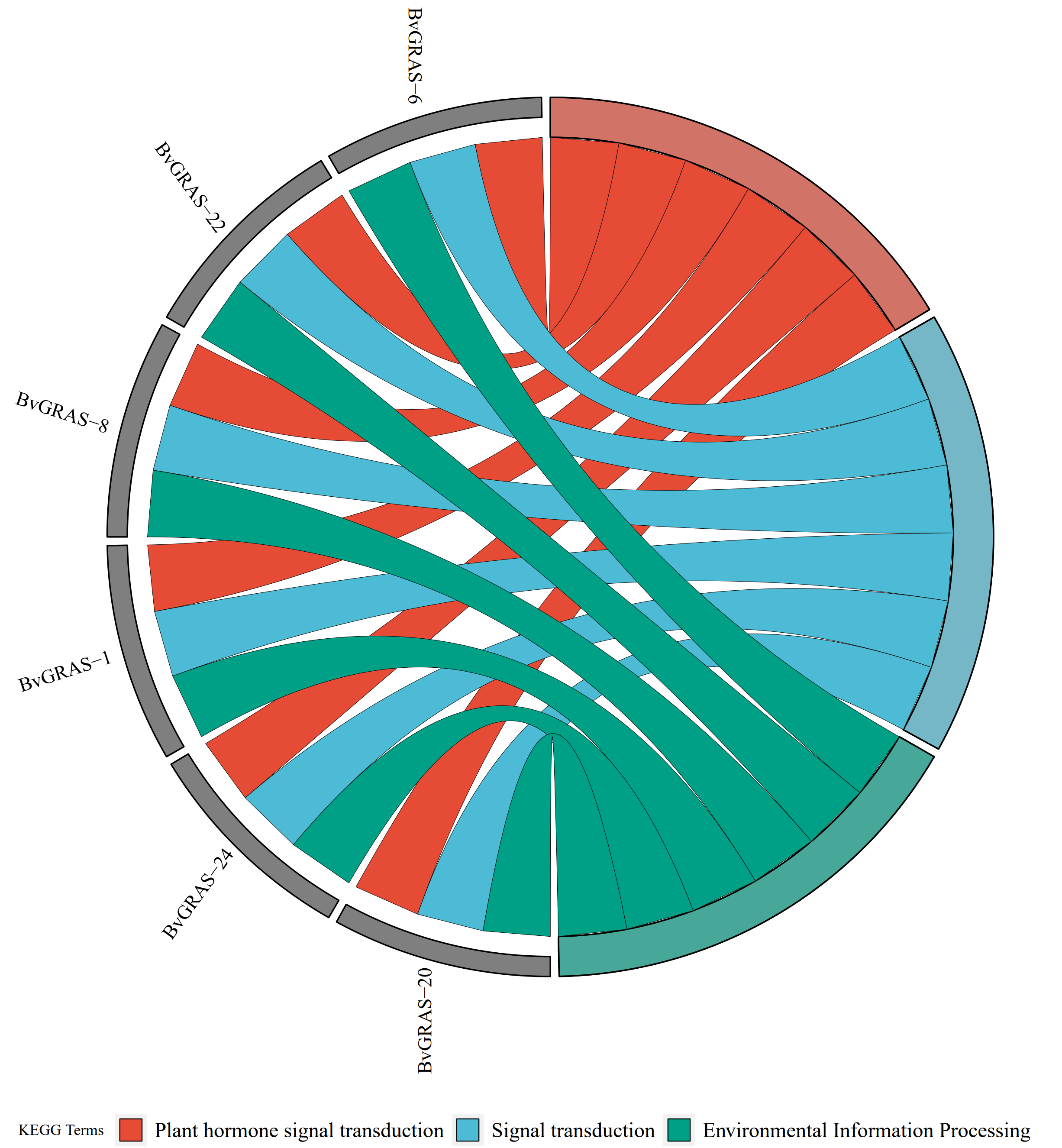

Supplement: Supplementary file 1 [file ijms-25-07132-s001.zip › Figure S4 KEGG enrichment analysis of BvGRAS TFs.png]

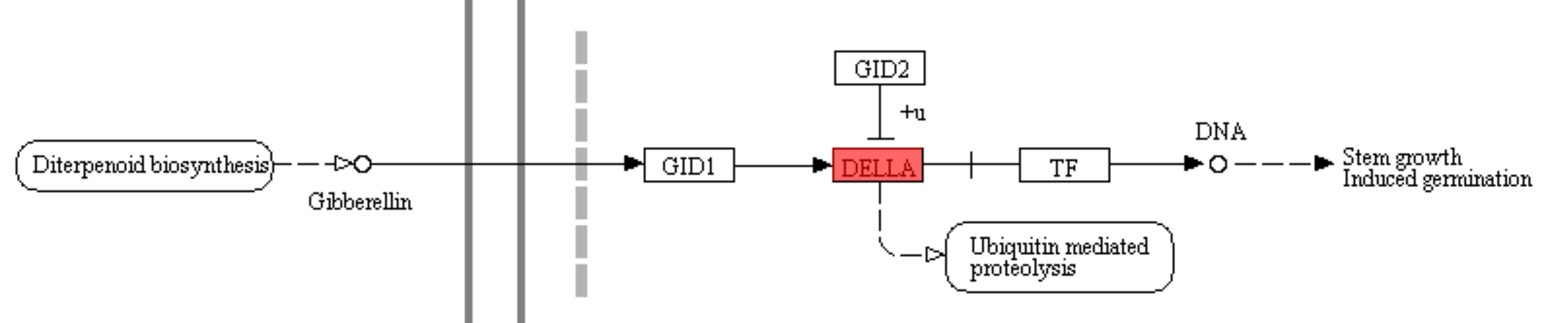

Supplement: Supplementary file 1 [file ijms-25-07132-s001.zip › Figure S5 The results of BvGRAS TF KEGG pathway analysis.png]

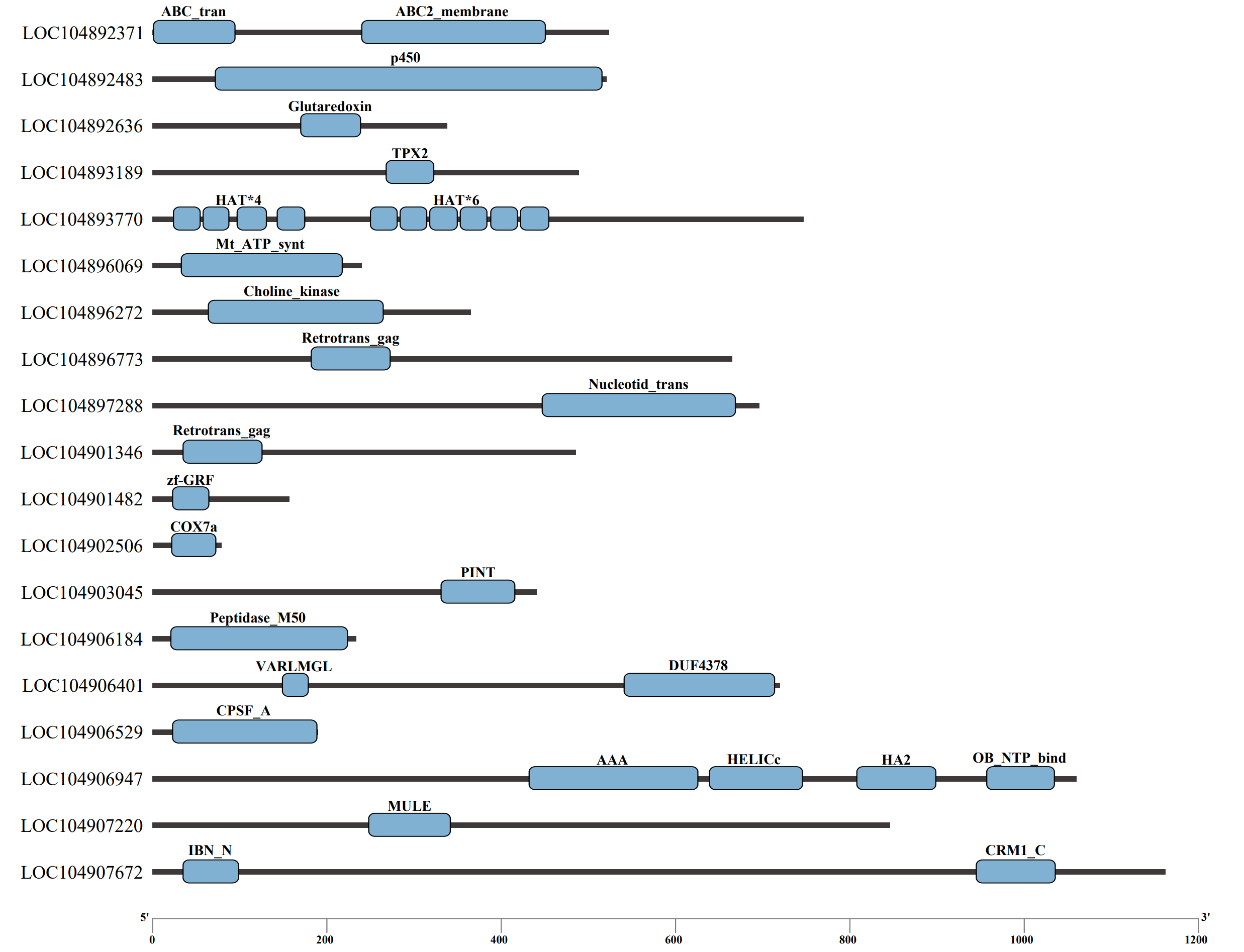

Supplement: Supplementary file 1 [file ijms-25-07132-s001.zip › Figure S6 The results of conserved domain analysis of target genes in BvGRAS regulatory network.png]

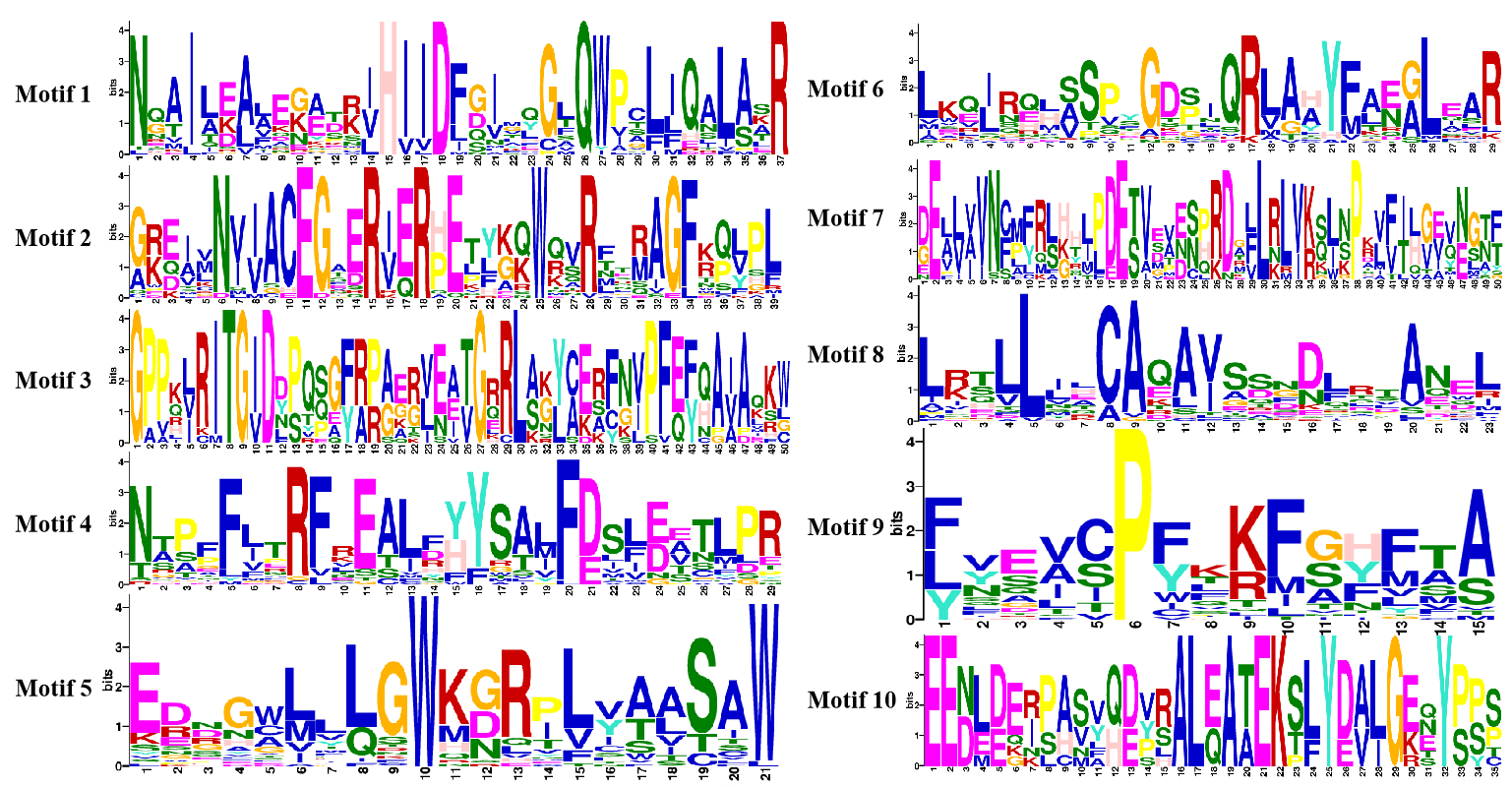

Supplement: Supplementary file 1 [file ijms-25-07132-s001.zip › Figure S1 BvGRAS motif sequences structure.png]
